# An Orthogonal Weighted Occupancy Likelihood Map with IMU-Aided Laser Scan Matching for 2D Indoor Mapping

**DOI:** 10.3390/s19071742

**Published:** 2019-04-11

**Authors:** Chuang Qian, Hongjuan Zhang, Jian Tang, Bijun Li, Hui Liu

**Affiliations:** 1 GNSS Research Center, Wuhan University, 129 Luoyu Road, Wuhan 430079, China; qc_gnss@whu.edu.cn (C.Q.); tangjian@whu.edu.cn (J.T.); loweliu@whu.edu.cn (H.L.); 2 State Key Laboratory of Information Engineering in Surveying, Mapping and Remote Sensing, Wuhan University, Wuhan 430079, China; lee@whu.edu.cn

**Keywords:** SLAM, orthogonal weighted occupancy likelihood map, IMU-aided laser scan matching, 2D indoor mapping

## Abstract

An indoor map is a piece of infrastructure associated with location-based services. Simultaneous Localization and Mapping (SLAM)-based mobile mapping is an efficient method to construct an indoor map. This paper proposes an SLAM algorithm based on a laser scanner and an Inertial Measurement Unit (IMU) for 2D indoor mapping. A grid-based occupancy likelihood map is chosen as the map representation method and is built from all previous scans. Scan-to-map matching is utilized to find the optimal rigid-body transformation in order to avoid the accumulation of matching errors. Map generation and update are probabilistically motivated. According to the assumption that the orthogonal is the main feature of indoor environments, we propose a lightweight segment extraction method, based on the orthogonal blurred segments (OBS) method. Instead of calculating the parameters of segments, we give the scan points contained in blurred segments a greater weight during the construction of the grid-based occupancy likelihood map, which we call the orthogonal feature weighted occupancy likelihood map (OWOLM). The OWOLM enhances the occupancy likelihood map by fusing the orthogonal features. It can filter out noise scan points, produced by objects, such as glass cabinets and bookcases. Experiments were carried out in a library, which is a representative indoor environment, consisting of orthogonal features. The experimental result proves that, compared with the general occupancy likelihood map, the OWOLM can effectively reduce accumulated errors and construct a clearer indoor map.

## 1. Introduction

Establishing an accurate and clear indoor map is a basic requirement of Indoor Navigation and Location-Based Services (INLBS). Mobile mapping is a highly efficient method for constructing an indoor map. Simultaneous Localization and Mapping (SLAM) is a popular and applicable method for mobile mapping in a GNSS-denied area, especially indoor environments [1,2].

SLAM estimates the pose and map in an unknown environment simultaneously [3]. According to the type of sensors, the SLAM techniques can be classified into vision-based and range-based approaches. Vision-based SLAM uses monocular, stereo or RGBD cameras to accomplish navigation and mapping and can obtain rich texture information [4,5,6,7,8]. While it is the cheapest approach, there are some disadvantages that limit its applications in indoor mapping, such as its sensitivity to lighting conditions, computational cost for processing large amounts of image data and the necessity for accurate calibration [9]. Range-based SLAM using sonar or laser sensors are applied in most indoor mapping techniques [9,10,11,12], because of its advantages of low complexity of data acquired and insensitivity to lighting conditions [9]. In this paper, Light Detection and Ranging (LiDAR) is utilized, which is one of the most popular ranging sensors, with a high ranging accuracy for SLAM [13,14], and which is utilized in many SLAM-based mapping applications [15,16].

There are two different major map representation methods with different positioning approaches. One of them is a feature-based map, in which the features of the indoor environment mainly include points and line segments. Point features can be detected by vision sensors, but for LiDAR, the line segment features are more detectable [1,9]. The positioning is completed using feature matching [1,17,18,19,20,21,22,23,24]. Feature extraction and association are the important steps for feature matching, which increases the complexity and may result in errors. Moreover, there may be no features in a laser scan. Compared to feature matching, the scan matching utilizes two or more consecutive frames of scan points directly for positioning. The classical scan matching algorithm is classical Iterative Closed Point (ICP) [25]. There are many improved algorithms, based on ICP, such as Polar Scan Matching (PSM) [26] and Iterative Closed Line (ICL) [27,28]. These methods calculate the relative position of consecutive pairs of scans, but the errors will accumulate rapidly over time. To overcome this problem, a grid-based occupancy likelihood map, which is an alternative method, is utilized to store the historical data, and scan-to-map matching is proposed to find the optimal position [29,30,31,32,33,34,35]. The key to this approach is how it generates the gird-based occupancy likelihood map. Scan-to-map matching is one of the most popular methods for indoor positioning and mapping, such as Hector SLAM [32] and Google’s Cartographer [34]. One of the scan-to-map matching algorithms with an occupancy likelihood map is Maximum Likelihood Estimation (MLE) [11,13,14,29,30]. In our work, we utilize MLE for scan-to-map matching.

While scan-to-map matching can achieve positioning directly, without feature extraction and association, some non-geometric or noise scan points produced by the objects, such as glass cabinets and bookcases, will affect the process involved in finding an optimal rigid-body transformation and decrease the accuracy. Moreover, the generalized occupancy likelihood map contains all the information collected by sensors, which includes useful structure information and noise information. It cannot clearly represent the indoor environment. In artificial environments, line segments are the principal elements and can provide considerable geometric information about the indoor environment. Using line features for positioning, the accuracy of localization will be enhanced [19]. Kuo et al. proposed a hybrid approach to enhance grid mapping by line matching and, for SLAM, utilized scan-to-map matching and line feature matching together [19]. Feature matching and scan matching still work independently. In fact, the line feature information could be utilized in generalizing the occupancy likelihood map. Then, scan-to-mapping can be enhanced, without extra computing complexity.

In the approaches using line segments, the parameters and covariance matrix of the line segments are calculated by the points that constitute a line segment [17,18,19,20,21,22,23]. This process compresses the data information of the scan points into the line parameter, which increases the computing complexity and may introduce errors, when the line segments are not very standard. It is better to use the points that constitute a line segment directly for mapping. The lines in most indoor environments are considered parallel or orthogonal to each other. This constraint is exploited to select line segments using various approaches in order to improve the robustness of the orientation and filter out many dynamic objects [21,22,23].

Inertial Measurement Units (IMUs) have been applied in many SLAM systems, primarily for attitude estimation [35,36,37]. The accurate attitude and orthogonal constraint can be combined to extract the orthogonal line segments efficiently. In this paper, a 2-D LiDAR and a commercial-grade IMU are utilized for 2D indoor mapping. An IMU-aided scan matching method is proposed for an orthogonal weighted occupancy likelihood map (OWOLM).

Compared with existing LiDAR-based indoor mapping solutions, this paper offers two major contributions. First, a new line segment extraction method is proposed, based on attitude-aided blurred segments, and this method combines the attitude and orthogonal constraint to extract the orthogonal quickly, without calculating the parameters of the lines. Second, the blurred segments are given a higher weight in generalizing the occupancy likelihood map, which can effectively reduce accumulated errors and construct a clearer indoor map. The remainder of this paper is organized as follows: Section 2 describes the methods utilized in this research; Section 3 introduces the indoor field tests and discusses the experimental results; and Section 4 draws conclusions.

## 2. Methods

### 2.1. Algorithm Overview

A common laser scan matching algorithm finds the optimal rigid-body transformation *T* that aligns the current laser scan *S_t_* at time *t* with the previous one *S*_*t*−1_ at time *t*−1. This method only considers two sequential laser scans, and when they are applied iteratively for all laser scans one by one, the pose drifting problem would deteriorate due to accumulated matching errors, which will affect the accuracy of the next matching.

The 2D space region of interest is gridded. The MLE is a grid-based and probabilistic scan matching method. It matches current scan *S_t_* to a grid-based occupancy likelihood map *M_t_*_−1_, instead of only matching pairs of scans *S_t_*_−1_ and *S_t_*. *M_t_*_−1_ is generated by all the previous scans, from 1 to *t*−1, and stores the likelihood value of each grid cell in the 2D space region. According to Bayes Rules, assuming the independence of each scan point of *S_t_*, the sum likelihood value of *S_t_* is computed as:

(1) $$P(S_{t}|M_{t-1})=\sum_{x\in S_{t}}^{}P(x|M_{t-1})$$

where *P*(*x*|M*_t_*_−1_) represents the probability that the scan point $x\in S_{t}$ drops in the *M_t_*_−1_ at that location. The current scan *S_t_* is matched against the map *M_t_*_−1_ by accumulating all the scan points. Due to the motion of our robotic platform, the LiDAR coordinate system varies over time as LiDAR’s dynamic position and attitude in the 2D space region. To match *S* against the map *M_t_*_−1_, we need to find the best rigid-body transformation $T^{*}$ from the LiDAR coordinate system of the current scan *S_t_* to that of the map *M_t_*_−1_, which is calculated by maximizing the likelihood value of the laser scan *S_t_* according to:

(2) $$T^{*}=\arg\max(P(T\propto S_{t}|M_{t-1})$$

where T ∝ *S_t_* is the set of *S_t_* laser points, transformed by the rigid body transformation T.

Therefore, there are two key issues to complete MLE-based scan matching: the generation of the occupancy likelihood map from all previous scans and an optimization framework to find the optimal rigid-body transformation. In terms of the first issue, some strategies were proposed to determine the likelihood value. One commonly used method is determining the likelihood value *P*(*x*|M*_t_*_−1_) by the distance *d*(*x*,F*_closest_*) of point $x\in S_{t}$ from the closest environment feature F*_closest_* using the Gaussian probability model:

(3) $$P(x|M_{t-1})\propto \exp(-d(x,F_{closest})/\sigma )$$

where σ is the standard deviation of the sensor measurement noise. In 2D indoor mapping by 2D LiDAR, the environmental features mean point features at intersections and line features (contours of walls, tables, cupboards, etc.). Several studies [11,14,31] used point features to calculate the likelihood value. The map stores all points from the previous scans. The distances of each point x in the current scan S*_t_* from the closest stored point x’ in the map are computed. Equation (3) is reconstructed as:

(4) $$P(x|M_{t-1})\propto \exp(-d(x,x\prime )/\sigma )$$

However, this approach probably fails in some situations, because it attempts to find correspondences for all points in the scans, even though a number of points may not correspond to the current scan. A contour is another feature of the main 2D structure of indoor environments, which is comprised of line segments [11,14]. In this contour-based approach, the contours are constructed by the pairs of adjacent points. Moreover, the grid cells, crossed by the contours, are considered to be the robust features. For the point x in the current scan *S_t_*, its occupy likelihood, along with the distance to the closest contour C, extracted from all previous scans, is calculated, which is similar to Equation (3):

(5) $$P(x|M_{t-1})\propto \exp(-d(x,C)/\sigma )$$

However, due to the influence of the measurement error, the contour information determined by two neighboring points may introduce a large error, as it might be a pseudo-contour. The line information should be extracted in a different way to calculate the likelihood value. In our work, orthogonal line segments are extracted after the optimal rigid-body transformation is found. Then, they are utilized in generating and updating the occupancy likelihood map.

In terms of the second issue, there are two main ways to find the optimal rigid-body transformation: brute search and gradient ascent methods. The gradient ascent method may get stuck at the local minimum, while the brute search method is a global search and is more robust. Moreover, a multi-resolutions map and narrow search window can greatly improve the search efficiency of the brute search method in time-expensive real-time application. Thus, the brute search method is selected in our work. In addition, the attitude and orthogonal constraint can be combined by fusing IMU and LiDAR, which improves both the efficiency and accuracy of scan matching. IMU can provide an accurate navigation solution, especially in relation to the attitude. Based on Tang’s platform [35], we utilized a 2D LiDAR and a commercial-grade IMU sensor, with MLE and the brute search method in an indoor environment, to achieve a highly accurate rigid-body transformation solution.

**Figure 1:**
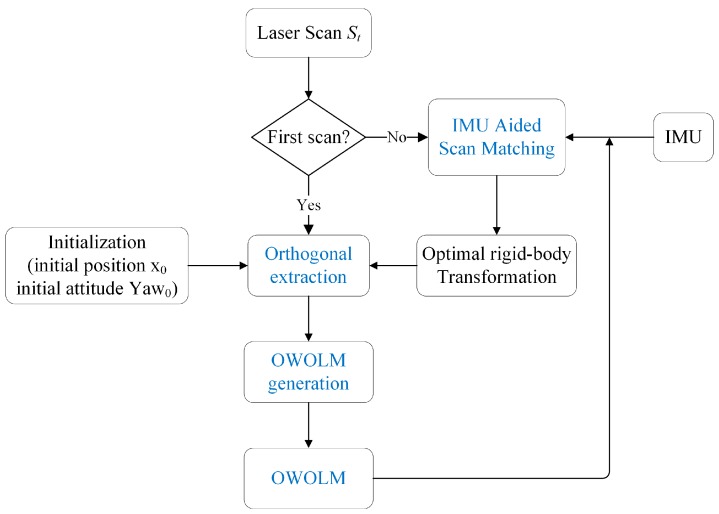
Flow chart of scan matching with OWOLM for indoor mapping.

The flowchart of our proposed SLAM algorithm is illustrated in Figure 1. Before the robotic platform starts to operate, we initialize its position and attitude for both IMU and LiDAR. During the operation, for the first laser scan *S*_1_ at time step 1, IMU-aided scan matching is skipped, as there is no prior map, but for all later laser scans, from time step 2, IMU-aided scan matching is applied to find the best rigid-body transformation to match *S_t_* to map *M_t_*_−1_, so that all the laser scans are projected to the coordinate system of LiDAR at time step 1 (base coordinate system). Secondly, after *S_t_* is transformed into the base coordinate system, orthogonal line segments are extracted. At last the OWOLM can be generated by the operation of 2-dimension Gaussian blurring, taking advantage of the orthogonal line segments. All the above steps are repeated to robustly achieve laser scans and the final OWOLM. Details of IMU-aided scan matching, orthogonal line segments extraction and the generation of occupancy likelihood map will be introduced in the following section.

### 2.2. IMU-aided Scan-to-Map Matching

IMU-aided Scan-to-Map Matching is based on an IMU and LiDAR fusion model. IMU can estimate the position, attitude and velocity of the system from the raw data: velocity and angular (pitch, roll and yaw) increments. Because of the drift of the accelerometer and gyroscope in IMU, the IMU outputs contain errors that cause the navigation results to rapidly drift. To solve this problem, an error propagation model, via first-order Taylor series expansion, is defined as follows:

(6) $$\delta r\;=\;[\delta p^{n},\delta v^{n},\delta \varepsilon^{n},\delta b_{a},\delta b_{g}]$$

(7) $$u=[\delta f^{b},\delta \omega_{}^{b}]$$

(8) $$\delta \overset{•}{r}=\mathrm{Fr}+\mathrm{Gu}$$

where *n* means the navigation coordinate system, which is north-east-down (NED), with its origin at the IMU sensor center in our work; and *b* is the body coordinate system, which is also defined at the IMU sensor center, with its *x*-axis positioning forward, *y*-axis, right and *z*-axis, down. δr is the error state, including the errors of position ($\delta p^{n}$), velocity ($\delta v^{n}$), attitude ($\delta \varepsilon^{n}$) at the navigation frame and drift of accelerometer ($\delta b_{a}$) and gyroscope ($\delta b_{g}$), $\delta f^{b}$ and $\delta \omega_{}^{b}$ are the white noise of the specific force, measured by an accelerometer, and body angular rate, measured by a gyroscope, respectively, and F and G are two matrices that vary with time (more details about F and G can be found in [38]). According to the theory of the linear system, Equation (8) is discretized in time as:

(9) $$\delta r_{t}=\Phi_{t|t-1}\delta r_{t-1}+\eta_{t-1}$$

(10) $$\Phi_{t|t-1}=\Phi (t-1,t)\approx e^{\int_{t-1}^{t}F(t)dt}\approx I+F_{t-1}\Delta t$$

(11) $$\eta_{t-1}\approx G_{t-1}W_{t-1}$$

where $\Phi_{t|t-1}$ is the state transition matrix, $\eta_{t-1}$ is the Gaussian distributed vector, I is the identity matrix, W*_t_*_−1_ is the driven response of the input white noise at time *t*−1, and Q reflects the standard deviation of IMU.

We use the MLE-based scan matching method, mentioned above, to get **r***_t_* and T* (calculated from x, y, yaw, which are from **r***_t_*) from LiDAR scans. Similar to Equation (3)/(4)/(5), here we do *t* calculate the distance, but instead find the grid cell in which the scan point locates and gets the likelihood value from the map *M_t_*_−1_ directly. Then, a brute search algorithm is applied for Equation (2) to estimate **r***_t_* and T*. For a large area, the brute search algorithm is time-consuming. To improve its efficiency, a multi-resolution map is used. The resolution of each fine grid-map is 4 times the preceding coarse one. The best estimation of **r***_t_*_−1_ and T* at the last time step is used as the initial value of the brute search algorithm for the next level, until the bottom one. Note that, using this strategy, we must know the likelihood map at all levels so that the process in Section 2.4 updates the map at all levels, which are used as the reference maps for the next incoming scan.

Equation (2) finds the T* that optimally matches the current scan to the reference map. To get a more accurate position, velocity, attitude and rigid-body transformation, we use an IMU and LiDAR fusion model, based on the Kalman Filter (KF). We put the above **r***_t_*, calculated by the MLE-based scan matching as **r***_t,Lidar_*, and **r***_t_*, from IMU motion mechanization as **r***_t,_*_IMU_. As IMUs’ frequency is higher than that of LiDARs, KF filters the results when there is LiDAR information **r***_t,Lidar_*. The difference between **r***_t,_*_IMU_ and **r***_t,Lidar_* is used as observation information $z_{t}$ in KF for $\delta r_{t}$ in Equation (9). By Equation (9), the prediction of $\delta r_{t}$, represented as $\delta r_{t}^{-}$, is calculated by $\delta r_{t-1}$. In EK, the state vector is updated by $z_{t}$:

(12) $$\delta r_{t}=\delta r_{t}^{-}+K_{t}(z_{t}-H_{t}\delta r_{t}^{-})$$

(13) $$P_{t}^{-}=\Phi_{t|t-1}P_{t-1}\Phi_{t|t-1}^{T}+Q_{t}$$

(14) $$K_{t}=P_{t}^{-}H_{t}^{T}{\left(H_{t}P_{t}^{-}H_{t}^{T}+R_{t}\right)}^{-1}$$

(15) $$P_{t}=(I-K_{t}H_{t})P_{t}^{-}$$

where K*_t_* is Kalman gain, H*_t_* the observation matrix which maps the state vector to observation vector, P*_t_*_−1_ and P*_t_* error covariance of state vector, $P_{t}^{-}$ the prior estimate of P*_t_*, R*_t_* the error covariance of observation vector. Finally:

(16) $$r_{t}=r_{t,\;\mathrm{IMU}}-\delta r_{t}$$

**r***_t_* includes the compensated position and attitude, from which the final T* can be calculated. **r***_t_* is also used as the initial value of the brute search for the next time step.

### 2.3. Orthogonal extraction by blurred segments

Commonly used line segmentation methods were summarized and compared in terms of their performances by Nguyen et al. [39], among which the Split-and-Merge method [40] and Incremental method [41] performed better than other methods [39] due to their high efficiency and accuracy. However, these two methods mainly use the residual test of line fitting for line segmentation and do not consider the orthogonal constraint in indoor environments. Moreover, some surfaces, such as book shelves in an indoor environment, are not smooth. Because of the unevenness of surfaces and the presence of noise, the blurred segment is an outstanding method for line segmentation.

**Figure 2:**
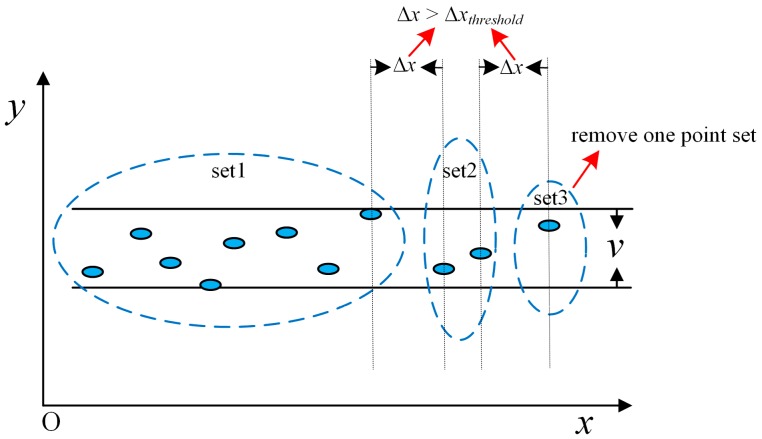
An illustration of OBS extraction for the *y*-coordinate.

The blurred segment is defined as a finite set of discrete two-dimensional points between line ax+by=μ and line ax+by=μ+w. The parameters of a blurred segment can be obtained by the support line of the convex envelope of this set of points [42]. If we consider the orthogonal constraint, after converting the current scan points according to the optimal rigid-body transformation, most of the line segments should be parallel to the coordinate axes, which means a=0 or b=0. The constraint blurred segment is called the orthogonal blurred segment (OBS). The blurred segments parallel to the *x* coordinate axe are determined by the points between line y=μ and line y=μ+v, and those parallel to the *y* coordinate axe are determined by the points between line x=μ and line x=μ+w. In our work, the width *v* equals *w*. To extract the OBSs with a certain width *v* or *w*, the sets of points whose x-coordinates vary less than *w*, or whose *y*-coordinates vary less than *v*, are selected. The OBS is more reliable when the set contains more points, so the set which contains the most points is chosen to be split and tested. Algorithm 1 shows the pseudo-code of the proposed OBS extraction algorithm for OBSs parallel to the *x* coordinate axis. The OBSs parallel to the *y* coordinate axe are extracted similarly. Figure 2 illustrates this process more vividly.

By the above process, OBSs can be quickly extracted only by the numerical comparison of coordinates. From Algorithm 1, we can see that the priori parameters include the width of OBS *v* or *w*, the minimum number n of the included points, the minimum segment length *l* and the maximum distance Δ*x_threshold_* between the adjacent points in OBSs. *v* or *w* is determined by the accuracy of ranging, which is usually 2*δ*, where *δ* is a ranging error, to guarantee that most of the scan points in the orthogonal can be contained in OBSs. *n* and *l* adapt to the environment and can be determined using a trial-and-error scheme.

Generally, *n* and *l* are larger, the OBS is more reliable, while some blurred segments would be missed. In the split process, Δ*x_threshold_* is determined by the range and angle sampling interval. We do not calculate the parameters from the points by line fitting. In contrast, we first determine the segmentation parameters and then find the points that constitute the OBS. This method can find the main line segments in an indoor environment quickly.

**Algorithm 1**: Pseudo-code of OBS extraction, where OBSs are parallel to *x* coordinate axeRequires: 1. Coordinates (*x*, *y*) of scan points 2. Width *v* of OBS 3. Minimum number *n* of points in OBS 4. Minimum length *l* of OBS, which is 1 in this paper 5. Maximum distance between the adjacent points in OBS Δ*x_threshold_*
sort the scan points according to the *y*-coordinates in ascending order for each point with *y*-coordinates *y*
collect the set of points in the *y*-coordinates between *y* and *y* + *v*
end for Find the set with the maximum number N of points if N > *n*
sort the set of points according to the *x*-coordinates in ascending order split the set if the adjacent *x*-coordinates Δ*x* >Δ*x_threshold_*
else exit the loop end if for each set if the length > *l*
save this set of points remove the point from the scan points end if end for

### 2.4. OWOLM Generation

**Figure 3:**
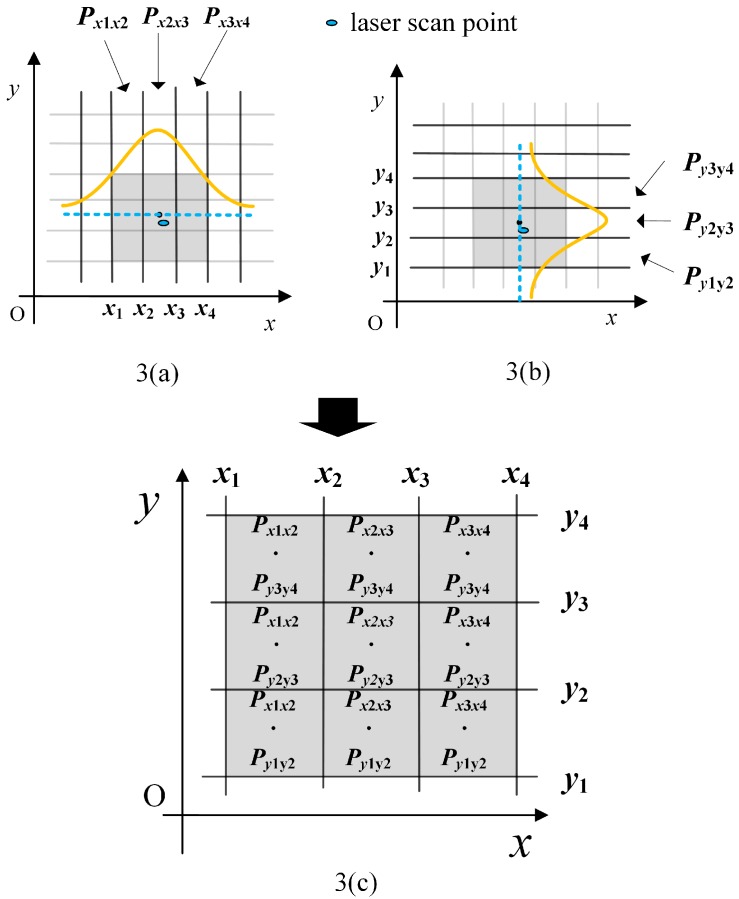
New occupancy likelihood values of nine grid cells around a laser point.

The occupancy likelihood map is the fundamental component for the scan-to-map matching algorithm. It provides the known background information, acquired from the previous laser scans. Grid-point occupation is a simple method to determine the likelihood value of each grid [13]. Tang et al. have combined this method and the contour-slope method and proposed a simple line-feature-based and three-level strategy [11]. These methods pre-define the likelihood value empirically, without extracting any features. In this paper, we propose a likelihood value determination method, according to the 2-D Gaussian blurring operation. Each point of a new laser scan is projected onto the map, after the optimal rigid-body transformation. The occupancy values of 9 grid cells around a laser point are calculated as Figure 3. In the figure, the black dot is the center point of the grid cell, which is closest to the scan point (represented by a blue ellipsoid). The occupancy likelihood is calculated by the x-coordinate (see 3(a)) and y-coordinate (see 3(b)) independently using the Gaussian assumption. In 3(a), grid cells in the first column have the same likelihood value, which is calculated by:

(17) $$P_{x_{1}x_{2}}=\frac{1}{\sigma_{x}\sqrt{2\pi }}\int_{x_{1}}^{x_{2}}\exp(-\frac{{\left(x-x_{c}\right)}^{2}}{2\sigma_{x}{}^{2}})dx$$

where $\sigma_{x}$ represents the standard deviation in the *x* axis, and $x_{c}$ is the x-coordinate value of the center grid. Similarly, $P_{y_{1}y_{2}}$ is calculated by:

(18) $$P_{y_{1}y_{2}}=\frac{1}{\sigma_{y}\sqrt{2\pi }}\int_{y_{1}}^{y_{2}}\exp(-\frac{{\left(y-y_{c}\right)}^{2}}{2\sigma_{y}{}^{2}})dy$$

Then, the new occupancy likelihood values of the nine grid cells are calculated, as in 3(c). If the blue ellipsoid shaped scan point is located in the OBS, mentioned above, $\sigma_{x}$ and $\sigma_{y}$ are set to much smaller values, which means that this scan point has a larger weight and higher probability of being located around grid cells.

**Figure 4:**
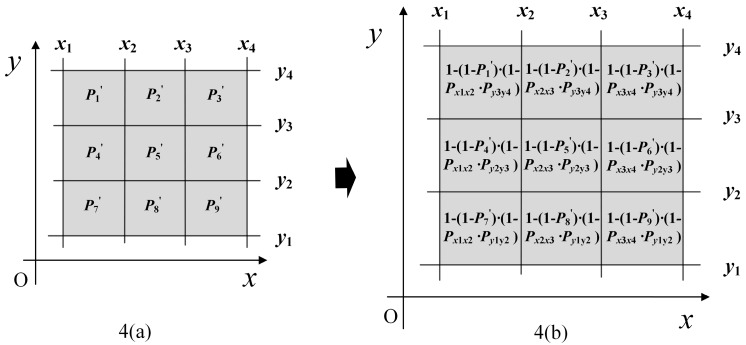
Updated occupancy likelihood values of the nine grid cells.

From *M_t_*_−1_, we get the prior occupancy likelihood values of these nine grid cells, marked as $P_{1}{}^{\prime }$, $P_{2}{}^{\prime }$ … $P_{9}{}^{\prime }$ in row-major order, as shown in Figure 4a. Then, the updated occupancy likelihood values of these 9 grid cells are calculated, as shown in Figure 4b. After all the scan points of the current scan at time step *t* update the *M_t_*_−1_ iteratively by repeating this process, a new OWOLM *M_t_* is generated.

## 3. Experimental Results

### 3.1. System overview

**Figure 5:**
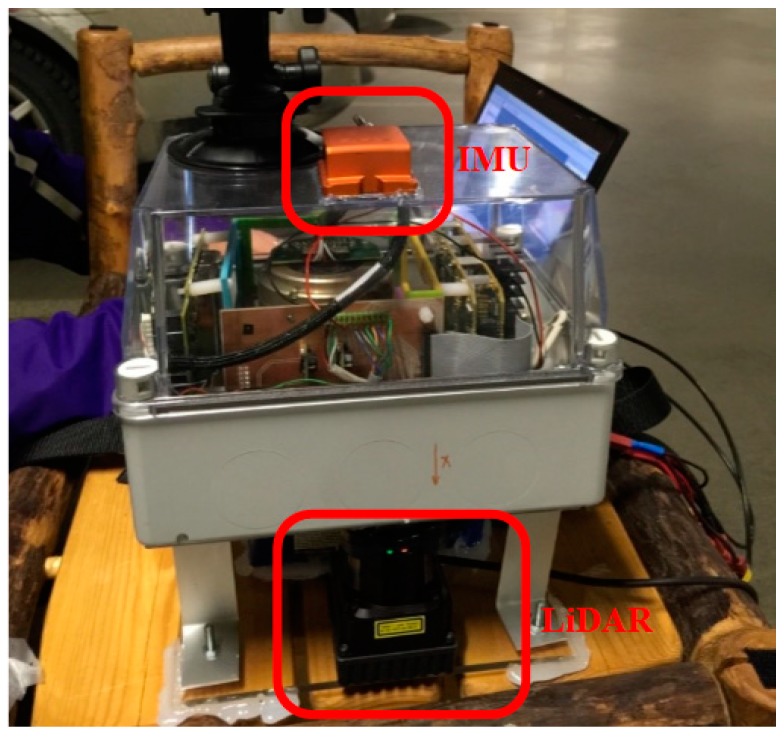
System hardware platform.

In order to verify the performance of the algorithm described in the following Section 3, a LiDAR/IMU integrated system (Figure 5) was designed and implemented. The measurement sensor LiDAR and Inertial Measurement Unit (IMU) are integrated on the mobile mapping platform to form the hardware of the system. A “UTM-30LX-EW” LiDAR, manufactured by Hokuyo Company (Osaka, Japan), was adopted for the platform. The LiDAR operated at 40 Hz and had a scanning angle range of 270 degrees, with an angular resolution of 0.25 degrees, a maximum effective range of 30 m and a range accuracy of ±30 mm at 0.1 m ~ 10 m and ±50 mm at 10 m ~ 30 m. The measurement accuracy of the LiDAR is in centimeters, and it is a medium precision device. The model of IMU is MTiG, which is a MEMS level device, and the bias stability of IMU gyroscopes is about 200.0 °/h, the bias stability of IMU accelerators is about 2000 mGal (1 Gal = 1 cm/s), and the sampling frequency of the IMU is 200 Hz.

### 3.2. Results

**Figure 6:**
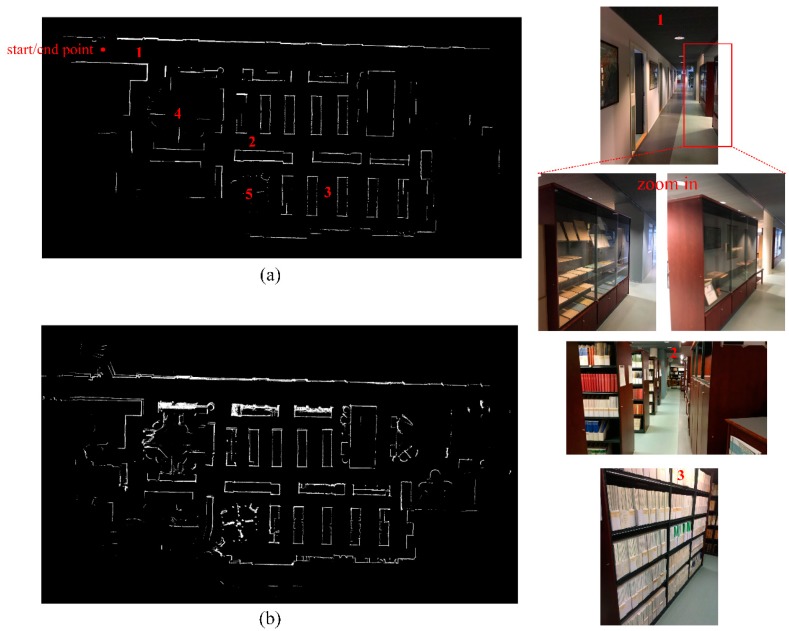
Comparative results of our proposed OWOLM (a), the traditional OLM (b) approach, and five scene photos of three places. In (a) and (b), brighter intensities indicate higher likelihood values.

Field tests were carried out in the library of the Finnish Geospatial Research Institute main building. Dynamic experiments along the aisles of the bookcases were conducted to evaluate the precision and effectiveness of our indoor mapping. Figure 6 shows the comparative results of the occupancy likelihood maps generated by the traditional OLM approach and our proposed OWOLM in a representative indoor scene. On the left, OWOLM (a) and OLM (b) are presented; on the right side, five scene photos of three places in the OWOLM are shown. In this test scene, there are many bookcases in the library and several glass cabinets in the corridor, as shown in the scene photos in Figure 6. Comparing Figure 6a,b, our OWOLM is much clearer and more distinct than the traditional OLM. More details on this will be discussed below.

The bookcases contain various layouts of books, with different shapes or empty shelves, which may introduce noise into indoor mapping. In our 2D map, bookcases are shown as rectangles, such as the perspectives from places number 2 and 3 in Figure 6. Comparing the mapping results of the bookcases in Figure 6a,b, we can see that, in our OWOLM approach the noise is reduced, and the contours of the bookcases are straighter, while the contours of the bookcases in 6(b) have more burrs or connection errors. This comparative result proves that our proposed method is more effective than the traditional OLM method, because it has far fewer connection errors in relation to contours.

In our test scene, in the place numbers 4 and 5 in Figure 6a, there are several tables and some scattered chairs around the tables. From the occupancy likelihood map in Figure 6b, we can see that, in this situation, the map generated by the traditional OLM approach looks messy and irregular. In our OWOLM, we used the orthogonal blurred segment, with the priori setting of parameters, the mapping of scattered tables and chairs, leaving out the unnecessary details and only keeping the main contours.

**Figure 7:**
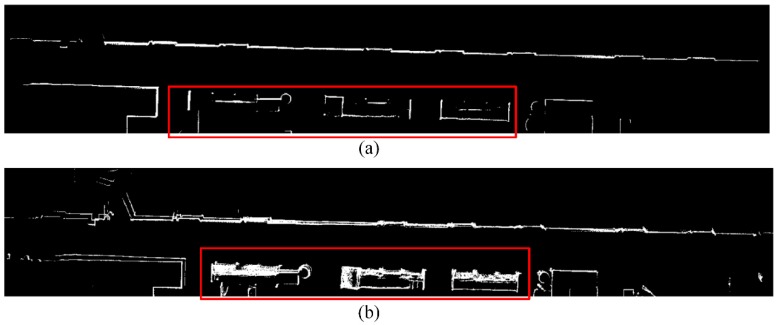
The occupancy likelihood maps of a long indoor corridor, with glass cabinets (shown in the red rectangles) on one side, generated by our proposed OWOLM (**a**) and the traditional OLM (**b**) approach. Brighter intensities indicate higher likelihood values.

Clear glass may also introduce errors into LiDAR by providing missing data or inaccurate data. Figure 7 is a zoom-in of place number 1 in Figure 6. We can obviously observe that the traditional OLM method is vulnerable to the negative effect of the glass. Many grid cells around the contours of glass cabinets have high, but wrong, occupancy likelihood values, with bright intensities. In contrast, our OWOLM method filters out LiDAR’s inaccurate data, caused by the glass, through orthogonal constraint, so that the contours of the glass cabinets are more distinct, and the mapping accuracy is improved.

Localization in the front-end of the SLAM system has accumulated errors. If the accuracy of localization is improved, the accumulated errors can be reduced, and the mapping precision can be further improved. In our experiment, we designed a loop trajectory, as shown in the Figure 6a. Our platform started and ended at the same point. If the accumulated error is small, the occupancy likelihood map, generated at the beginning of the trajectory, should coincide well with the map produced in the end phase of the mapping. 

**Figure 8:**
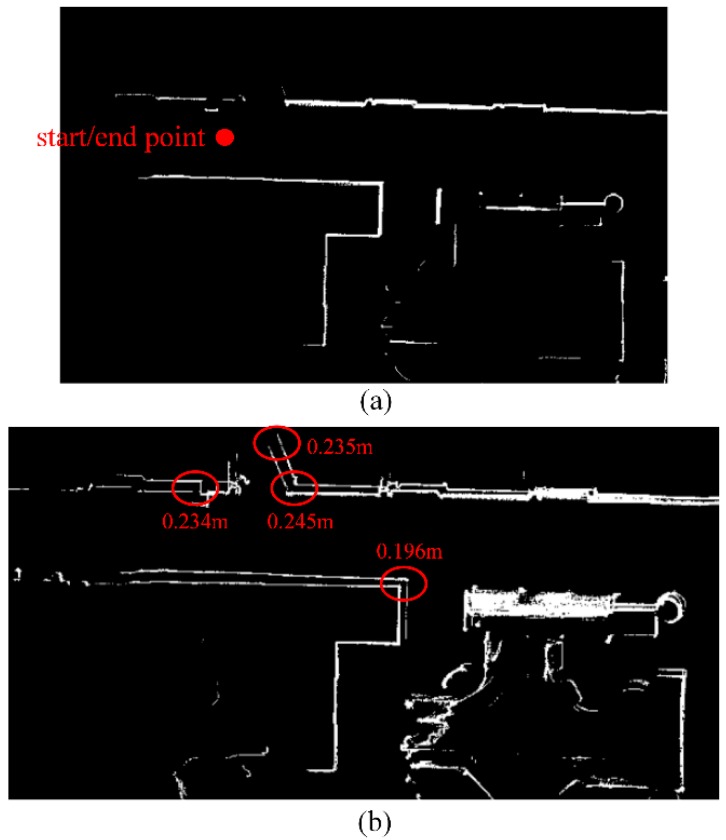
The occupancy likelihood maps near the start/end point, generated by our proposed OWOLM (**a**) and the traditional OLM (**b**) approach. Brighter intensities indicate higher likelihood values. Red numbers represent biases between two contours in the red ellipsoid.

**Figure 9:**
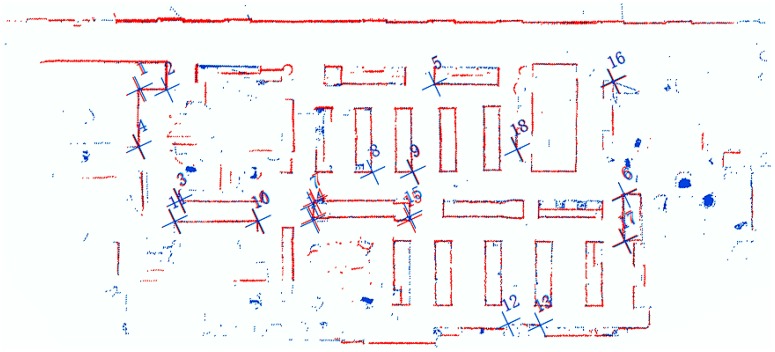
The occupancy likelihood maps, generated by our platform (red) and TLS (blue) in the TLSs’ coordinate frame. Cross marks and numbers represent 18 correspondence feature points.

Figure 8 shows a zoom-in of the mapping results near the start/end point. In Figure 8b, there is an obvious bias in the occupancy likelihood maps from the two mapping phases. We analyzed the mapping data at four places in Figure 8b and found that the accumulated error in the localization achieved a sub-meter level. However, in Figure 8a, the bias is largely decreased. The noise scan points, produced by objects, such as glass cabinets and bookcases, are filtered out by our OWOLM method, and the IMU and LiDAR fusion model is used, so the localization precision in the front-end is largely improved, and the mapping accuracy is improved as well. To further evaluate the performance of our OWOLM method, we used a Terrestrial Laser Scanner (TLS) to measure and map our test scene, as a reference. The mapping accuracy of TLS is at the millimeter level and can evaluate our mobile mapping results using the OWOLM approach. 18 feature points are chosen to analyze the mobile mapping results. Using the coordinates of the 18 correspondence feature points in OWOLM’s coordinate frame and TLS’s coordinate frame, the transformation parameters of the two coordinate frames is calculated. Three transformation parameters are considered: translation along the *X* and *Y* axis and rotation. The transformation parameters transform the coordinates of the 18 feature points from OWOLM’s coordinate frame to TLS’s coordinate frame, as shown in Figure 9. The residual is calculated using the difference in the coordinates between TLS’s coordinate frame in the *X* (residual_x) and *Y* (residual_y) direction and the Euclidean distance (residual), as shown in Table 1. We can see that most of the points have residuals of only 1cm or 2cm or 3cm.

## 4. Discussion

In many cases, such as in a large area, closed-loop detection is difficult or unavailable. The accumulated error of the front-end in SLAM could be reduced by closed-loop detection in the back-end of SLAM. In our experiments, we did not use closed-loop detection but an IMU and LiDAR fusion model.

Our OWOLM approach, based on IMU-aided scan-to-map matching and orthogonal blurred segments, can largely improve the localization and mapping accuracy of SLAM. It can filter out the inaccurate scan points, caused by bookcases and glass, such as glass cabinets or windows, from laser scans. Additionally, our IMU and LiDAR fusion model also improved the attitude and position estimation. Due to its theoretical basis, our proposed IMU-aided OWOLM approach is suitable for indoor environments, where orthogonal features are rich. Fortunately, the main indoor structures have abundant orthogonal features, such as walls, doors, desks, cupboards and so on.

In the process of generating orthogonal blurred segments, parameters are defined before mapping. These parameters may affect the results of segments and the final OWOLM. For example, different maximum distances between adjacent points would generate line features of different lengths. Therefore, these parameters are empirical and vary, depending on the indoor environment.

## 5. Conclusions

Based on the data, collected by a 2D laser scanner and IMU mounted on a mobile platform, a new indoor SLAM algorithm via scan-to-map matching, aided by the grid-based occupancy likelihood map, was proposed in this paper. Instead of matching two sequential laser scans to find the optimal rigid body transformation, we used IMU-aided scan-to-map matching, which was based on an IMU and LiDAR fusion model, to alleviate the accumulated matching error. First, the scan-to-map matching method, based on the occupancy likelihood map and a brute search algorithm, was proposed to find a rough transformation, which was combined with IMU motion mechanization by a Kalman filter to get an optimal transformation. Then, a blurred segment was applied to extract the orthogonal in the indoor environment. A 2-D Gaussian blurring operation was applied to update the likelihood value of the occupancy likelihood map, where orthogonal blurred segments gained higher weights. Finally, the orthogonal feature weighted occupancy likelihood map (OWOLM) was generated. Field test results, in several data sets, acquired from a typical indoor environment, demonstrated that our proposed SLAM algorithm can provide high-precision positioning and mapping results.

## Figures and Tables

**Table 1:** The residual error (cm) of the 18 feature points. Residual_x and residual_y are residual errors of the x-coordinate and y-coordinate, respectively, and residual is the root mean square of residual_x and residual_y.

No.	1	2	3	4	5	6	7	8	9	10	11	12	13	14	15	16	17	18
residual_x	5.8	−1.8	2.5	2	1.4	1.9	−12.5	−0.6	−1.6	2.9	1	0.3	1.3	−8.5	6.2	−1	3.5	−2.7
residual_y	−1.5	0.1	1.5	0.4	2.2	−4.7	−6.7	0.3	1.7	−1.1	0.5	1.5	2.6	−7.4	14.5	1.8	−6.2	0.7
residual	6	1.8	2.9	2.1	2.6	5	14.2	0.7	2.3	3.1	1.1	1.6	2.9	11.3	15.7	2.1	7.2	2.8
